# *Forsythia suspensa* Protects against Bone Loss in Ovariectomized Mice

**DOI:** 10.3390/nu11081831

**Published:** 2019-08-08

**Authors:** Youn-Hwan Hwang, Seon-A Jang, Taesoo Kim, Hyunil Ha

**Affiliations:** Herbal Medicine Research Division, Korea Institute of Oriental Medicine, Yuseong-daero 1672, Yuseong-gu, Daejeon 34054, Korea

**Keywords:** *Forsythia suspensa*, osteoporosis, ovariectomy, osteoclast differentiation

## Abstract

In traditional oriental medicine, the fruit of *Forsythia suspensa* has been used as a nutritional supplement to alleviate inflammation and treat gastrointestinal diseases. However, there is no information available on its beneficial effects on bone. We investigated the beneficial effects of *F. suspensa* water extract (WFS) on osteoclast differentiation and bone loss. The microarchitecture of trabecular bone was analyzed by micro-computed tomography. Osteoclast differentiation was evaluated based on tartrate-resistant alkaline phosphatase activity, and bone resorption activity was examined on a bone-like mineral surface. The mechanism of action of WFS was assessed by evaluating the expression and activation of signaling molecules. Phytochemical constituents were identified and quantitated by ultrahigh-performance liquid chromatography–tandem mass spectrometry. WFS reduced ovariectomy-induced trabecular bone loss and inhibited receptor activator of nuclear factor-κB ligand (RANKL)-induced osteoclast formation and resorption activity. WFS suppressed RANKL-induced expression of nuclear factor of activated T cells cytoplasmic 1, a crucial transcription factor for osteoclast differentiation by decreasing c-Fos protein levels and suppressing the activation of p38 and c-Jun-N-terminal kinase. We also identified 12 phytochemicals in WFS including lignans, phenylethanoids, and flavonoids. Collectively, these results suggest that WFS inhibits osteoclast differentiation and can potentially be used to treat postmenopausal osteoporosis.

## 1. Introduction

Bone remodeling is a tightly regulated process that balances bone formation by osteoblasts and bone resorption by osteoclasts. In postmenopausal osteoporosis, an imbalance in bone remodeling—especially an elevation in osteoclast activity—leads to deterioration of bone architecture, low bone mineral density, and reduced bone strength [1]. Osteoclasts are multinucleated bone-resorbing cells that arise via proliferation, differentiation, and fusion of hematopoietic progenitor cells. Receptor activator of nuclear factor (NF)-κB ligand (RANKL) is a key cytokine inducing osteoclast differentiation that activates NF-κB and mitogen-activated protein kinase (MAPK) signaling pathway components including inhibitor of κB (IκB), p38, extracellular signal-regulated kinase (ERK), and c-Jun-N-terminal kinase (JNK), as well as nuclear factor of activated T-cells cytoplasmic 1 (NFATc1), a master transcription factor for osteoclast differentiation [2,3]. Given the critical role of osteoclasts in bone disorders, therapeutic strategies that attenuate excess osteoclast formation and resorption activity may be an effective treatment for osteoporosis.

Although several standard therapies are available for the treatment of osteoporosis, there is a need for safer but nonetheless effective alternatives. Natural products and herbal medicines are gaining interest for their potential to improve bone health in osteoporosis patients and healthy individuals [4]. The fruit of *Forsythia suspensa*, a widely distributed plant in Eastern Asia and Europe, has anti-pyretic and anti-inflammatory activities and is used to treat infectious diseases in traditional oriental medicine; its anti-oxidant, anti-bacterial, anti-viral, and anti-inflammatory properties have been demonstrated in modern pharmacological studies [5]. *F. suspensa* also has beneficial effects against disorders of lipid and glucose metabolism [6], and a previous study showed that the fruit, branch, and leaf extract of *F. koreana* inhibited osteoclast formation and bone resorption activity [7]. However, there is little information on the anti-osteoporotic properties of *F. suspensa*.

To address this issue, the present study investigated whether the water extract of *F. suspensa* (WFS) can alleviate osteoporosis using a mouse model of ovariectomy-induced bone loss as well as the underlying mechanisms. To date, about 250 phytochemicals with various biological activities have been found in fruit of *F. suspensa*, including alkaloids, lignans, phenylethanoids, terpenes, flavonoids, and steroids [5,8]. Characterizing these bioactive phytochemicals can clarify the molecular basis for the anti-osteoporotic activity of WFS. In this study, the phytochemical profile of WFS was analyzed by ultrahigh-performance liquid chromatography–tandem mass spectrometry (UHPLC–MS/MS).

## 2. Materials and Methods

### 2.1. Materials

α-Minimal essential medium (α-MEM), fetal bovine serum (FBS), acetonitrile, water, and formic acid were purchased from Thermo Fisher Scientific (Waltham, MA, USA). WFS was obtained from the National Development Institute of Korean Medicine (Gyeongsan, Korea) and stored in the herbarium (voucher number # JW-17) of the Korean Institute of Oriental Medicine. Briefly, dried WFS (0.5 kg) was extracted with distilled water (3.5 L) under reflux for 3 h and lyophilized after filtration. The brownish WFS powder was stored at −20 °C until use in experiments.

### 2.2. Animal Study and Micro-Computed Tomography (Micro-CT) Analysis

Animal studies were approved by the Institutional Animal Care and Use Committee of Knotus (Guri, Korea). Female C57BL/6 mice (6 weeks old) were obtained from Japan SLC (Shizuoka, Japan) and acclimated for 7 days under standard housing conditions prior to experiments. The mice had free access to water and feed. Mice were sham-operated (control group; *n* = 5) or ovariectomized (OVX) via bilateral dorsal incision under Zoletil 50 (Virbac, Carros, France) and Rumpun (Bayer, Leverkusen, Germany) anesthesia. OVX mice were randomly divided into three groups (*n* = 5 each) that were treated with vehicle (OVX/vehicle), a low dose of WFS (OVX/WFS L; 200 mg/kg/day), or a high dose of WFS (OVX/WFS H; 500 mg/kg/day). Starting on day 7 post-surgery, the mice were fed with a high-fat diet (HFD; 60 kcal%; Research Diet, New Brunswick, NJ, USA), and a standard chow diet (Cargil, Pyengtaek, Korea) were provided before feed exchange. WFS was administered by oral gavage once daily for 4 weeks. Gonadal fat pads and femurs were dissected following euthanasia with an overdose of Zoletil and Rumpun. For histological analysis, the femurs were fixed with 10% phosphate-buffered formalin and decalcified, embedded in paraffin, and stained with hematoxylin and eosin.

The microarchitecture of the femur was analyzed using a Quantume GX μCT imaging system (PerkinElmer, Hopkinton, MA, USA). The distal femur was scanned with the following settings: 90 kV, 88 mA, and a 14 min scanning time. The scanned images were reconstructed, and trabecular bone parameters were analyzed using SkyScan software (Bruker, Kontich, Belgium) as previously reported [9]. Bone volume to tissue volume ratio (BV/TV), trabecular number (Tb.N), trabecular separation (Tb.S), and trabecular thickness (Tb.Th) were determined.

### 2.3. Osteoclast Differentiation and Resorption Assay

Bone marrow-derived macrophages (BMMs), prepared according to a previously described method [10], were cultured in α-MEM containing 10% FBS and 30 ng/mL macrophage colony-stimulating factor (M-CSF). The BMMs were seeded at 1 × 104 cells/well in a 96-well plate or 1 × 105 cells/well in a 6-well plate and treated with M-CSF and RANKL (100 ng/mL, provided by Dr. Yongwon Choi (University of Pennsylvania) for 4 days to induce osteoclast differentiation in the absence or presence of WFS. Osteoclast differentiation was evaluated based on tartrate-resistant acid phosphatase (TRAP) activity by TRAP staining and colorimetric analysis according to published methods [10].

To assess resorption activity, BMMs (1.5 × 104 cells/well) were seeded in Osteo assay plates (Corning Inc., Corning, NY, USA) and treated with M-CSF and RANKL in the absence or presence of WFS for 4 days. After removing the cells by using a 5% sodium hypochlorite solution, resorption pits were observed under the microscope. The relative area of resorption was measured using ImageJ software (National Institutes of Health, Bethesda, MD, USA).

### 2.4. Real-Time Quantitative PCR

Total RNA and cDNA were prepared using the RNeasy kit (Qiagen, Hilden, Germany) and High-Capacity cDNA Reverse Transcription Kit (Thermo Fisher Scientific), respectively, according to the manufacturers’ instructions. Quantitative PCR was performed using the TaqMan Universal PCR Master Mix (Applied Biosystems, Foster City, CA, USA) and TaqMan primers for c-Fos (Mm00487425_m1), NFATc1 (Mm00479445_m1), and interferon (IFN)-β (Mm00439552_s1) on an ABI 7500 Real-Time PCR system (Applied Biosystems). Target mRNA levels were calculated and normalized to that of 18S rRNA (Hs99999901_s1).

### 2.5. Western Blotting

Whole cell lysates were extracted using a radioimmunoprecipitation assay lysis buffer containing protease and phosphatase inhibitors, and protein concentration was measured using a bicinchoninic acid assay kit (Thermo Fisher Scientific). Equal amounts of protein were resolved by sodium dodecyl sulfate–polyacrylamide gel electrophoresis and transferred to a polyvinylidene difluoride membrane that was incubated for 1 h with primary antibodies (1:1000 dilution) followed by secondary antibodies (1:2000 dilution). Antibodies against p38, phospho-p38, ERK, phospho-ERK, JNK, phospho-JNK, p65, and phospho-p65 were obtained from Cell Signaling Technology (Danvers, MA, USA). Antibodies against NFATc1, c-Fos, and β-actin along with secondary antibodies were from Santa Cruz Biotechnology (Santa Cruz, CA, USA). Protein bands were detected with a chemiluminescence reagent, and signal intensity was measured using the ChemiDoc imaging system (Bio-Rad, Hercules, CA, USA).

### 2.6. UHPLC–MS/MS Analysis

Twelve reference standards of phytochemicals found in WFS were purchased from ChemFace (Wuhan, China). The phytochemical constituents in WFS were identified and quantitated using a Dionex UltiMate 3000 system coupled with a Thermo Q-Exactive mass spectrometer as previously described [11,12]. An Acquity BEH C18 column (100 × 2.1 mm, 1.7 μm) with 0.1% formic acid in water and acetonitrile was used, and gradient elution was performed. The Q-Exactive mass spectrometer was equipped with a heated electrospray ionization source and operated in the positive and negative ion-switching modes. The spectrum was obtained in full MS and data-dependent MS_2_ scan mode. The full acquisition parameters were as follows: Resolution, 70,000; scan range, 100–1500 *m*/*z*; and the profile spectrum type. The MS_2_ parameters were as follows: Resolution, 17,500; loop count, 10; normalized collision energy, 25; and the centroid spectrum type. Data acquisition and analysis were performed using Xcalibur and Tracefinder software (Thermo Fisher Scientific).

### 2.7. Statistical Analysis

Data are presented as mean ± standard error of the mean (SEM) for the in vivo study and mean ± standard deviation (SD) for the in vitro study. Statistical analysis was performed by one-way analysis of variance with Dunnett’s post hoc testing using Prism (Graphpad, San Diego, CA, USA). A *p*-value less than 0.05 was considered statistically significant.

## 3. Results and Discussion

### 3.1. WFS Alleviates OVX-Induced Bone Loss

**Figure 1:**
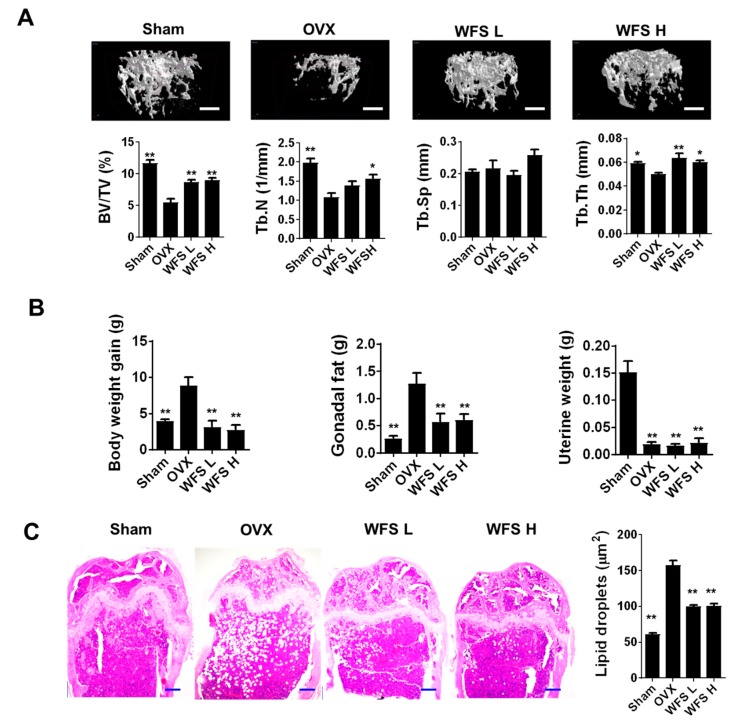
Inhibition of bone loss and lipid accumulation by *Forsythia suspensa* water extract (WFS) in ovariectomized (OVX) mice. (**A**) Micro-CT analysis of distal femur (scale bar, 0.5 mm) and uterine weights. (**B**) Bodyweight gain during experimental period, gonadal fat weight, and uterine weight. (**C**) Histopathological analysis of femur by hematoxylin and eosin staining (scale bar, 250 μm) and the average size of lipid droplets in the bone marrow. Sham, sham-operated/vehicle; OVX, OVX/vehicle; WFS L, OVX/low-dose WFS treatment (200 mg/kg/day); WFS H, OVX/high-dose WFS treatment (500 mg/kg/day). BV/TV, bone volume to tissue volume ratio; Tb.N, trabecular number; Tb.Sp, trabecular separation; Tb.Th, trabecular thickness. Data are expressed as mean ± SEM (n = 5). * *p* < 0.05, ** *p* < 0.01 vs. OVX only.

OVX in mice has been criticized that it leads to a rapidly hormonal interruption in place of a gradual decline, which naturally occurs in peri- and post-menopausal women [13]. However, OVX in mice is a popular experimental model in drug development for osteoporosis despite its limitation, because OVX causes bone loss by enhancing bone remodeling through estrogen deficiency, similar to post-menopausal osteoporosis [14]. Moreover, OVX mice fed with an HFD, as an animal model of post-menopausal metabolic syndrome, are characterized for alteration of body weight gain and fat accumulation in adipose tissues [15]. Increased fat accumulation in bone marrow aggravates bone loss in estrogen deficiency-related osteoporosis, and is correlated with increased fracture risk in humans [16,17]. Recently, it has been shown that marrow adipocyte accumulation impairs bone healing and regeneration [18]. Morphological examination of bone microarchitecture can provide critical information on the degree of bone loss under pathophysiological conditions. In the present study, we investigated the beneficial effects of WFS (200 and 500 mg/kg/day) on bone in OVX mice fed with an HFD through micro-CT analysis for bone microarchitecture and histopathological examination for fat accumulation. Representative micro-CT scans of the distal femur trabecular region in each group are shown in Figure 1A. OVX caused bone loss and uterine hypotrophy compared with the sham group (Figure 1A,B), consistent with a previous report [19]. However, mice treated with WFS showed compaction and thickening of the cancellous bone relative to OVX mice without affecting the uterine hypotrophy, indicating that WFS alleviates OVX-induced bone loss independently of estrogenic effects. A morphometric analysis of trabecular bone revealed that WFS mitigated the OVX-induced decreases in BV/TV, Tb.N, and Tb.Th (*p* < 0.05 or *p* < 0.01). In addition to bone loss, OVX also increased body weight, gonadal fat weight, and fat deposits in bone marrow compared with sham-operated animals (*p* < 0.01), which was significantly attenuated by WFS (*p* < 0.01) (Figure 1B,C). These results indicate that WFS can attenuate estrogen deficiency-induced bone loss and fat accumulation, suggesting that WFS might be a useful candidate for the treatment of obesity and osteoporosis in postmenopausal women.

### 3.2. WFS Inhibits Osteoclast Differentiation and Resorption Activity

**Figure 2:**
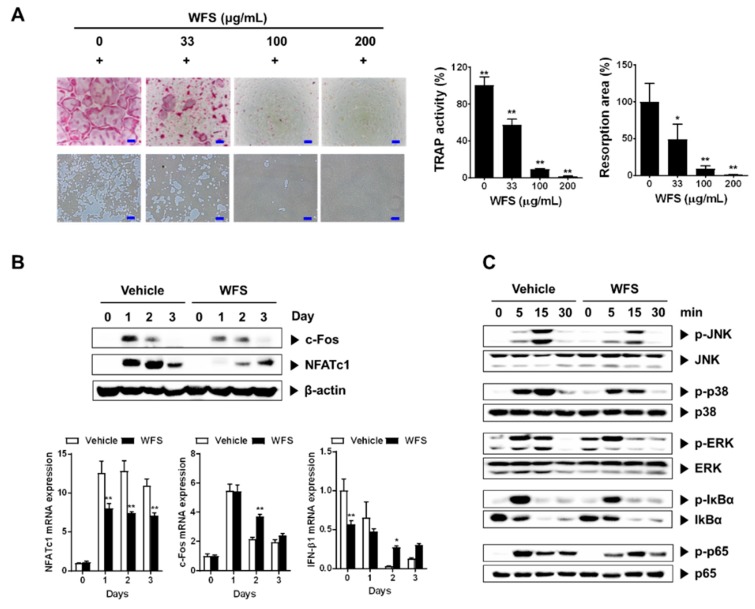
Inhibitory effects of WFS on RANKL-induced osteoclast differentiation of bone marrow-derived macrophages (BMMs). (**A**) Inhibition of osteoclast differentiation by WFS. Tartrate-resistant acid phosphatase (TRAP) staining (left, top), resorption pits (left, bottom), TRAP activity (middle), and resorption area (right). (**B**) Inhibitory effects of WFS on the expression of c-Fos and NFATc1. BMMs were pretreated with vehicle (distilled water) or WFS (100 μg/mL) for 3 h and then treated with RANKL (100 ng/mL) for the indicated days. The protein and mRNA levels were determined by Western blot and real-time PCR analyses, respectively. (**C**) Modulatory effects of WFS on RANKL-induced early signaling pathways. BMMs were pretreated with vehicle or WFS (100 μg/mL) for 3 h and then treated with RANKL (100 ng/mL) for the indicated times. Cell lysates were subject to Western blot analysis. Data are representative mean ± SD of three independent experiments. * *p* < 0.05, ** *p* < 0.01 vs. control treated with vehicle. p-JNK, phospho-JNK; p-p38, phospho-p38; p-ERK, phospho-ERK; p-p65, phospho-p65; p-IκBα, phospho-IκBα.

Having established that WFS has a beneficial effect on bone loss, we next focused on the effects of WFS on osteoclasts, mainly responsible for bone loss. In the presence of M-CSF, RANKL induces the differentiation of osteoclast precursors such as BMMs into osteoclasts [20,21]. WFS inhibited the formation of TRAP-positive multinucleated osteoclasts in M-CSF- and RANKL-treated BMMs in a dose-dependent manner (Figure 2A). TRAP activity, which is widely used as a marker of osteoclast maturation, was also reduced by WFS at all concentrations tested. Similar to the results, WFS decreased pit formation caused by the resorption activity of osteoclasts.

To clarify the mechanism of the inhibitory action of WFS, we evaluated the expression of NFATc1, a key transcription factor for osteoclastogenesis, and its upstream transcription factor c-Fos [22,23]. Treatment of BMMs with WFS downregulated RANKL-induced NFATc1 mRNA and protein expression, whereas it suppressed RANKL-induced c-Fos protein levels but not mRNA levels (Figure 2B). Previously, it has been shown that IFN-β negatively regulates osteoclast differentiation by inhibition of c-Fos protein expression without affecting RANKL-induced c-Fos mRNA levels [24]. Thus, we examined IFN-β expression. WFS did not affect IFN-β expression in BMMs on one day after stimulation of RANKL while suppressing c-Fos protein expression (Figure 2B), suggesting its action independently of IFN-β production. We next investigated whether WFS modulates RANKL-mediated activation of MAPK and NF-κB, which are the early signaling pathways that are activated during osteoclast differentiation. RANKL treatment resulted in the activation of p38, ERK, JNK, and NF-κB in BMMs within 15 min (Figure 2C). However, WFS suppressed the RANKL-induced phosphorylation of p38 and JNK while having no effect on that of ERK, NF-κB/p-65, and IκBα. JNK can phosphorylate and activate c-Fos as a member of the activator protein-1 transcription factor family, and p38 is involved in the expression of c-Fos and NFATc1 during RANKL-induced osteoclast differentiation [25,26]. Collectively, our results suggest that WFS suppresses RANKL-induced NFATc1 expression by decreasing c-Fos protein levels and suppressing the activation of p38 and JNK, thereby inhibiting osteoclast differentiation and bone loss. However, the molecular mechanisms by which WFS downregulates RANKL-induced c-Fos protein levels and NFATc1 expression remain to be clarified.

### 3.3. Phytochemical Constituents of WFS

**Figure 3:**
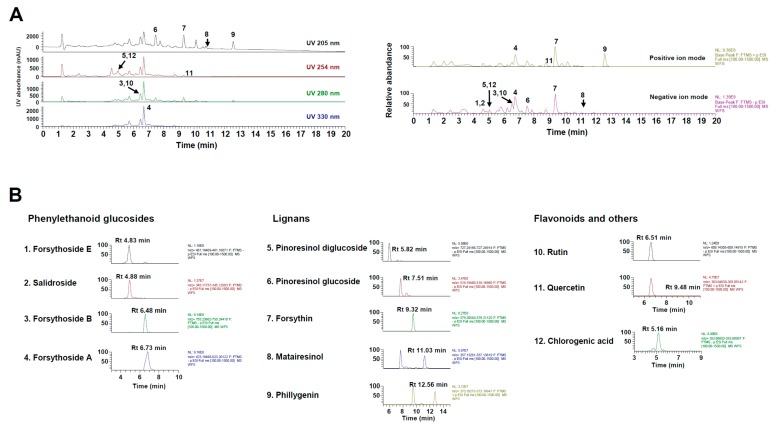
Ultrahigh-performance liquid chromatography–tandem mass spectrometry (UHPLC–MS/MS) analysis of WFS. (**A**) Ultraviolet and base peak chromatograms. (**B**) Extracted ion chromatogram of identified phytochemicals. UV, ultraviolet; Rt, retention time.

Characterizing the phytochemical constituents of herbal medicine-derived products can provide important information on their biological activities [27]. UHPLC–MS/MS analysis of WFS identified four phenylethanoid glucosides (i.e., forsythoside A, forsythoside B, forsythoside E, and salidroside), five lignans (i.e., forsythin, matairesinol, phillygenin, pinoresinol glucoside, and pinoresinol diglucoside), two flavonoids (i.e., quercetin and rutin), and chlorogenic acid by comparison with the mass spectra and retention times of authentic standards (Figure 3 and Table 1). All phytochemicals identified in this study have been previously reported [5,8], and their relative abundance in WFS was about 7.91% (*w*/*w*). Phenylethanoid glucosides were more abundant than the other phytochemical groups. Chlorogenic acid, forsythoside A, pinoresinol diglucoside, quercetin, rutin, and salidroside have been shown to exert anti-osteoporotic and anti-osteolytic effects in animal models [28,29,30,31,32,33], and other studies have reported the inhibitory effects of chlorogenic acid, forsythoside A, matairesinol, quercetin, and rutin on osteoclast differentiation [28,30,34,35]. However, forsythoside B, forsythoside E, forsythin, phillygenin, and pinoresinol glucoside have not been reported in bone metabolism. Thus, the anti-osteoporotic effects of WFS may be attributed to the pharmacological properties of the identified components, although further studies are needed to elucidate their mechanisms of action and contribution to the efficacy of WFS.

## 4. Conclusions

This is the first study to demonstrate the beneficial effects of WFS on bone health in an estrogen-deficient state. We showed that WFS inhibited osteoclast differentiation and suppressed estrogen deficiency-induced bone loss and lipid accumulation in bone marrow. We also identified 12 phytochemical constituents with potential anti-osteoporotic activity. These findings provide evidence that WFS is an effective naturally derived alternative to standard therapies for the treatment of postmenopausal osteoporosis. The mechanisms underlying its inhibitory effect on lipid accumulation in bone marrow remains to be further studied.

## Figures and Tables

**Table 1:** Phytochemical constituents of WFS by UHPLC–MS/MS.

No.	R_t_ (Min)	Calculated (*m*/*z*)	Estimated (*m*/*z*)	Adducts	Error (ppm)	Formula	MS/MS Fragments (*m*/*z*)	Identifications	Amounts (mg/g)
1	4.83	461.1665	461.1669	[M − H]^−^	−1.925	C_20_H_30_O_12_	315.1086, 205.0710, 135.0438	Forsythoside E	5.628
2	4.88	345.1191	345.1192	[M + HCO_2_]^−^	1.035	C_14_H_20_O_7_	299.1138, 119.0336	Salidroside	0.690
3	6.48	755.2404	755.2402	[M − H]^−^	−0.251	C_34_H_44_O_19_	623.1979, 461.1671, 161.0231	Forsythoside B	0.301
4	6.73	623.1981	623.1983	[M − H]^−^	−0.913	C_29_H_36_O_15_	623.1979, 461.1671, 179.0339, 161.0231	Forsythoside A	62.625
5	5.82	727.2455	727.2457	[M + HCO_2_]^−^	0.640	C_32_H_42_O_16_	357.1342, 151.0388	Pinoresinol diglucoside	0.089
6	7.51	519.1872	519.1874	[M − H]^−^	−0.231	C_26_H_32_O_11_	357.1342, 151.0388	Pinoresinol glucoside	4.773
7	9.32	579.2083	579.2084	[M + HCO_2_]^−^	−0.768	C_27_H_34_O_11_	371.1501, 356.1261	Forsythin	0.875
8	11.03	357.1344	357.1345	[M − H]^−^	−0.860	C_20_H_22_O_6_	357.1343, 313.1441, 221.0814, 137.0595	Matairesinol	0.137
9	12.56	373.1646	373.1649	[M + H]^+^	−0.171	C_21_H_24_O_6_	341.1379, 165.0546, 151.0390	Phillygenin	2.268
10	6.51	609.1461	609.1465	[M − H]^−^	−0.524	C_27_H_30_O_16_	301.0346, 300.0275	Rutin	1.658
11	9.48	303.0499	303.0498	[M + H]^+^	0.387	C_15_H_10_O_7_	303.0499	Quercetin	0.005
12	5.16	353.0878	353.0878	[M − H]^−^	−0.445	C_16_H_18_O_9_	191.0553	Chlorogenic acid	0.051

Rt, retention time; ppm, part per million.
